# Case 3/2018 - A 60-year-old Female with Chagasic Heart Disease, Admitted Due to Heart Failure Decompensation, Cachexia and Pulmonary Infection

**DOI:** 10.5935/abc.20180100

**Published:** 2018-07

**Authors:** Gustavo Alonso Arduine, Vera Demarchi Aiello

**Affiliations:** Instituto do Coração (InCor) do Hospital das Clínicas da Faculdade de Medicina da Universidade de São Paulo (HC-FMUSP), São Paulo, SP - Brazil

**Keywords:** Chagas Disease, Chagas Cardiomyopathy, Heart Failure, Cachexia, Pneumonia

The patient is a 60-year-old female with Chagas heart disease and cachexia, admitted due to heart failure decompensation attributed to bronchopneumonia. She eventually had a cardiac arrest with pulseless electrical activity after lung biopsy.

The patient was being followed up at InCor since the age of 48 years, diagnosed with Chagas disease. She initially complained of palpitations, which subsided after amiodarone was prescribed. In addition, she had systemic arterial hypertension.

Her laboratory tests revealed: potassium, 5.2 mEq/L; sodium, 144 mEq/L; creatinine, 0.8 mg/L; hemoglobin, 16.2 g/dL; hematocrit, 48%; glycemia, 87 mg/dL; cholesterol, 200 mg/dL; triglycerides, 53 mg/dL; TSH, 1.16 microIU/mL; free T4, 1.1 ng/dL; ALT, 8 IU/L; AST, 10 IU/L.

At the time, the ECG revealed diffuse ventricular repolarization changes.

The echocardiogram (August 2004) showed the following: aorta, 28 mm; left atrium, 52 mm; septal thickness, 11 mm; posterior wall, 7 mm; left ventricle (diast/syst), 71/62 mm; left ventricular ejection fraction (LVEF), 26%, with posterior (basal), inferior (basal) and lateral (basal) akinesia, and small apical aneurysm; right ventricle, 28 mm (dilated and hypokinetic); severe mitral regurgitation; and right ventricular systolic pressure, 65 mm Hg.

The chest X-ray (2012) showed cardiomegaly (Figure 1).

**Figure 1:**
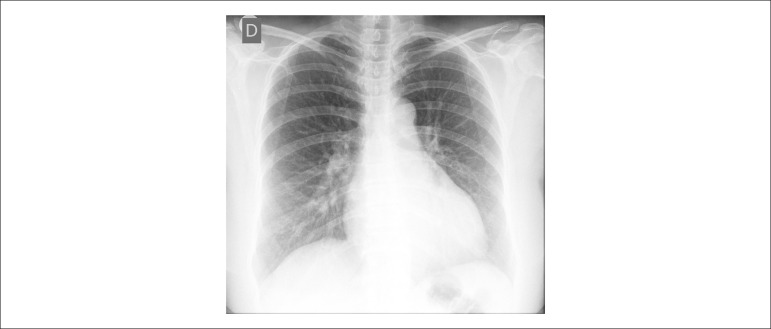
Chest X-ray (posterior-anterior view): increased pulmonary vasculature and cardiomegaly (+++).

The Holter ECG at that time showed frequent ventricular extrasystoles and nonsustained ventricular tachycardia.

 The patient remained asymptomatic until 2013 (57 years of age) using hydrochlorothiazide (25 mg), spironolactone (25 mg), carvedilol (12.5 mg), enalapril (20 mg) and amiodarone (100 mg) daily. In April 2013, she had a resuscitated cardiac arrest, preceded by malaise and sustained ventricular tachycardia, receiving an implantable cardioverter defibrillator (ICD) with cardiac pacemaker programing for bradycardia episodes (ICD-T - ICD with antibradycardia and antitachycardia pacing and shock), being prescribed 600 mg of amiodarone daily.

Five days before that episode, she had upper digestive bleeding with hematemesis, when endoscopy revealed gastric ulcer with no active bleeding, and clean base (Forrest III), with a lower risk for rebleeding.

The patient experienced appropriate shock in May 2014. She was receiving an amiodarone dose lower than prescribed, thus the dose was increased, but the patient did not tolerate it because of dyspepsia. A cardiac electrophysiology study was indicated, aimed at the possible ablation of the sustained ventricular tachycardia pathways.

The chest X-ray revealed pulmonary congestion and more severe cardiomegaly (Figure 2).

**Figure 2:**
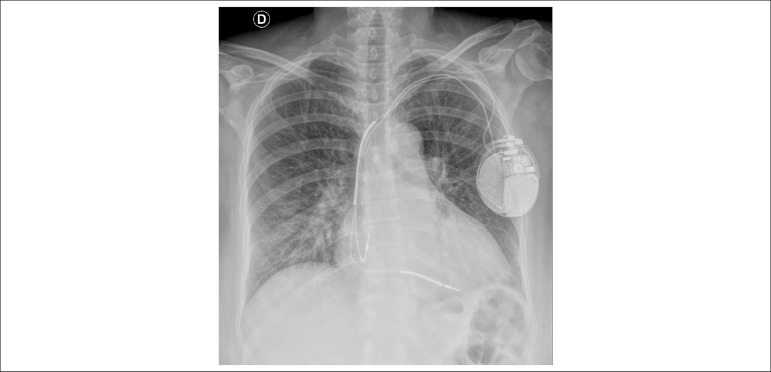
Chest X-ray (posterior-anterior view): presence of cardiac pacemaker, worse pulmonary congestion, cardiomegaly (+++).

The echocardiogram (August 4, 2014) revealed: aorta, 27 mm; left atrium, 43 mm; ventricular septal thickness, 9 mm, posterior wall, 8 mm; left ventricular diameters, 68/57 mm; LVEF, 30%. The left ventricle showed eccentric hypertrophy and reduced systolic function due to an inferolateral wall aneurysm (mid and basal segments) and an apical aneurysm. The right ventricle was normal. There was moderate mitral valve regurgitation. The pulmonary artery pressure was 25 mm Hg.

Her coronary tomography angiography (July 28, 2014) evidenced no coronary lesion. The cardiac electrophysiology study (July 31, 2014) identified poorly-tolerated sustained ventricular tachycardia triggered by extra-stimuli, which required electric cardioversion. The electroanatomic mapping revealed a scar associated with low and slow late potentials in the lateral (mid and basal segments), antero-lateral (mid and basal segments) and inferolateral (mid and basal segments) walls. Because of the proximity to the circumflex sub-branches, the radiofrequency pulses were not delivered through the epicardium, but through the endocardium. After the procedure, the new stimuli no longer triggered ventricular tachycardia similar to that initially observed. However, several poorly tolerated tachycardias of different morphologies were triggered, requiring cardioversion.

On outpatient follow-up (November 2014), the patient complained of dyspnea on exertion and dizziness when changing from supine to orthostatic position.

Her physical examination (November 11, 2014) revealed: weight, 70 kg; height, 1.7 m; arterial blood pressure, 102/80 mmHg; heart rate, 68 bpm. The pulmonary auscultation was normal. On cardiac auscultation, her heart rhythm was regular with no abnormal heart sound, and a systolic heart murmur was heard over the mitral area (++/4+). The abdominal examination was normal. There was no edema, and her pulses were palpable and symmetrical. Because of her complaints and arterial blood pressure levels, hydrochlorothiazide was suspended, and the other medications, maintained.

Her laboratory tests (October 2014) showed: hemoglobin, 13.2 g/dL; hematocrit, 43%; leukocytes, 7220/mm^3^ (normal differential count); platelets, 200000/mm^3^; total cholesterol, 180 mg/dL; HDL-cholesterol, 73 mg/dL; LDL-cholesterol, 88 mg/dL; triglycerides, 95 mg/dL; glycemia, 94 mg/dL; creatinine, 0.96 mg/dL; sodium, 141 mEq/L; potassium, 4.8 mEq/L; AST, 79 IU/L; ALT, 111 IU/L; uric acid, 4.2 mg/dL; C-reactive protein, 6.78 mg/L; TSH, 2.85 µg/mL; free T4, 1.62 mg/dL.

On outpatient follow-up visits (October 2015 and March 18, 2016), she denied dyspnea, chest pain, palpitations and syncope, but complained of dizziness. The ICD/pacemaker assessment was normal.

Her chest X-ray (2015) revealed pulmonary congestion and cardiomegaly (Figure 3).

**Figure 3:**
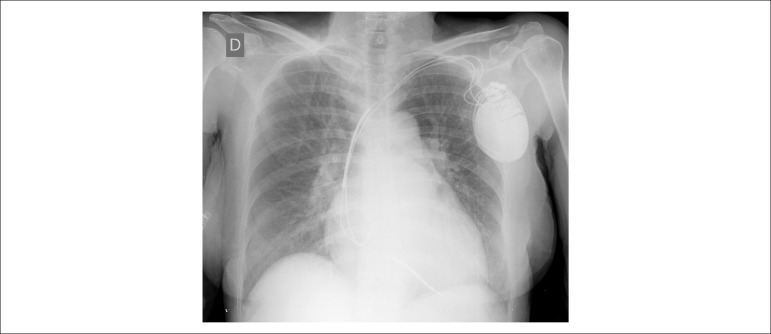
Chest X-ray (posterior-anterior view): increase and cephalization of the pulmonary vasculature.

On September 22, 2016, the patient was admitted due to decompensated heart failure and bronchopneumonia. She reported progressive worsening of dyspnea, which was then triggered on mild exertion. She denied chest pain and fever, but reported dry cough, lack of appetite and weight loss. Her physical examination revealed an emaciated patient (53 kg), on regular general condition, tachypneic, with heart rate of 72 bpm, arterial blood pressure of 95/60 mm Hg, and O_2_ saturation of 92%. Her auscultation revealed rales over the pulmonary bases, rhythmic heart sounds, and systolic heart murmur over the mitral area (++++/6+). Her abdomen was flaccid, with a tender liver, palpated 2 cm from the costal margin. There was lower limb edema (+++/4+).

The patient was using amiodarone (200 mg), enalapril (5 mg), hydrochlorothiazide (25 mg), levothyroxine (25 µg), metoprolol (100 mg), simvastatin (20 mg), warfarin (5 mg).

Her ECG (September 22) showed: sinus rhythm; heart rate, 63 bpm; indirect signs of right atrial overload (Peñaloza-Tranchesi); low-voltage QRS complexes on the frontal plane; probable electrically inactive lateral area; left anterior-superior hemiblock (Figure 4). After a few days, the new ECG revealed an operational pacemaker with atrial stimulus propagating to the ventricle (AAI) (Figure 5).

**Figure 4:**
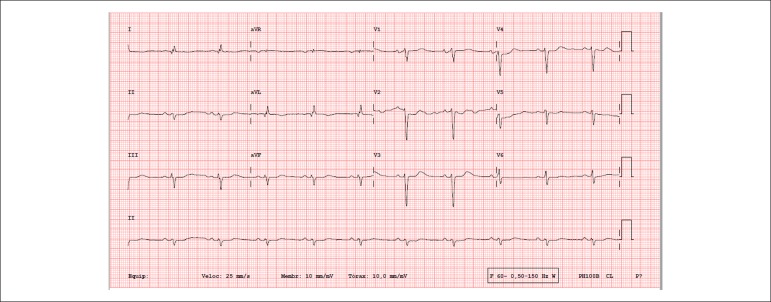
ECG: sinus rhythm, low-voltage QRS complexes on the frontal plane, probable electrically inactive lateral area, right bundle-branch block, left anterior‑superior hemiblock.

**Figure 5:**
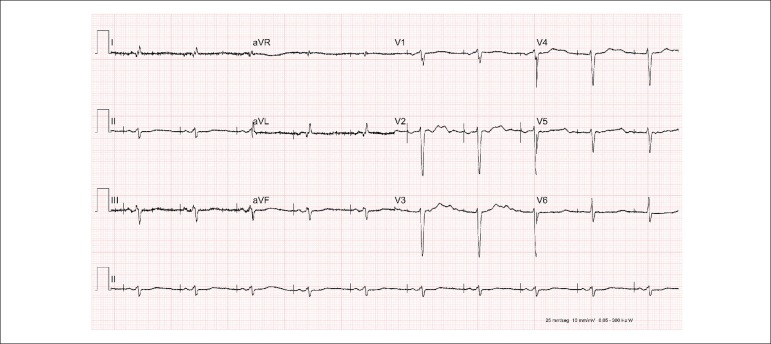
ECG: operational pacemaker with atrial stimulus propagating normally to the ventricle.

Her chest X-ray (September 22, 2016) evidenced the presence of an ICD/pacemaker with electrodes in the left atrium and ventricle, pulmonary congestion, opacity area suggestive of pneumonia in the right pulmonary lower field (air bronchogram), elevation of the left main bronchus (suggestive of enlarged left atrium), and global enlargement of the heart area, attributed mainly to the right ventricle (Figure 6).

**Figure 6:**
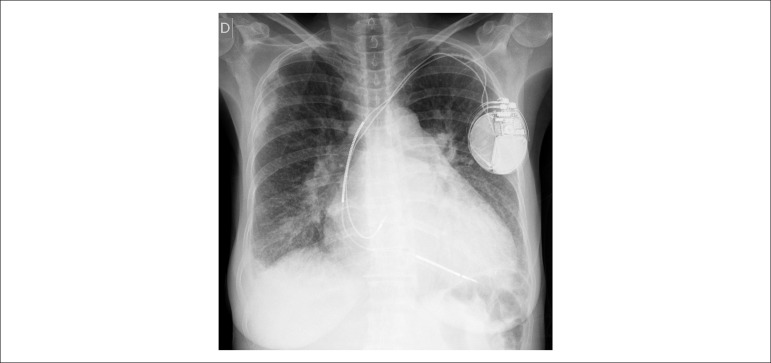
Chest X-ray (posterior-anterior view): presence of cardiac pacemaker, pulmonary congestion, and cardiomegaly (++++/4).

Her laboratory tests (September 22) revealed: hemoglobin, 8.2 g/dL; hematocrit, 26%; leukocytes, 17500/mm^3^ (neutrophils 79%, eosinophils 0%, lymphocytes 15% and monocytes 6%); platelets, 344000/mm^3^; urea, 33 mg/dL; creatinine, 0.71 mg/dL; AST, 148 IU/L; ALT, 136 IU/L; gamma GT, 36 IU/L; alkaline phosphatase, 75 mg/dL; total proteins, 6.9 g/dL; albumin, 3.1 g/dL; C-reactive protein, 124.39 mg/L; sodium, 140 mEq/L; potassium, 3.6 mEq/L. Arterial blood gas analysis: pH, 7.54; pCO_2_, 31.1 mm Hg; pO_2_, 62.3 mmHg; O_2_ saturation, 93%; bicarbonate, 26.2 mmol/L; base excess, 3.9 mmol/L.

Because of the diagnostic suspicion of pneumonia, ceftriaxone and clarithromycin were initiated.

The dyspnea and edema improved, arterial hypotension episodes occurred, and the dry cough and mild hyperthermia (37.6ºC) persisted.

Assessment of the ICD/pacemaker revealed 14 episodes of ventricular tachycardia in July 2016: 12 abolished by burst (high-frequency stimuli) and 2 abolished with 31-J shocks.

Her echocardiogram (September 26, 2016) showed: aorta, 26 mm; left atrium, 60 mm; basal and mid right ventricle, 43 mm and 33 mm, respectively; ventricular septal thickness, 10 mm; posterior wall, 7 mm; left ventricle (diast/syst), 73/60 mm; LVEF, 40%. The atria were severely enlarged, the left ventricle was dilated with dyskinesia of the lateral wall (basal segment), akinesia of the inferior wall (basal segment), and apical aneurysm. The right ventricular function was normal. The mitral and tricuspid valves showed severe regurgitation due to inadequate leaflet coaptation. Systolic pulmonary artery pressure was estimated at 47 mmHg. No pericardial change was observed.

On the days following admission, her laboratory tests continued to show leukocytosis (around 19000), anemia, hypoalbuminemia (1.9 g/dL) and elevated C-reactive protein (above 150 mg/L).

A new chest X-ray detected an opaque nodule in the right base (Figure 7).

**Figure 7:**
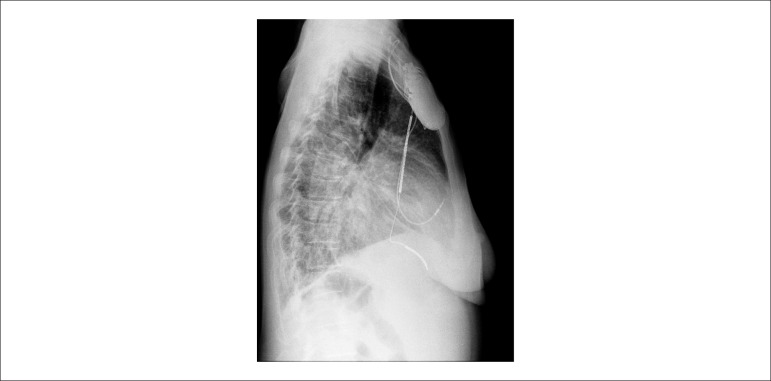
Chest X-ray (lateral view): similar to the previous ones, the only difference being the presence of an opaque nodule in the right base.

Chest tomography (September 28, 2016) evidenced: pacemaker with endocavitary electrodes in the right chambers; dilation of the pulmonary trunk (41 mm); permeable trachea and main bronchi of normal caliber; diffuse thickening of the bronchial walls; enlarged pulmonary hila; and hilar calcifications that could be lymph node sequelae. There was an irregular nodule measuring 2.3 x 2.5 x 1.9 cm in the transition between the medial and lateral segments of the middle lobe, in addition to diffuse ground-glass opacity with septal thickening, mainly in the bases, compatible with congestion. Furthermore, there was a peripheral hyperdense area, close to the pleural surface of the right superior lobe, suggestive of subacute parenchymal bleeding or accumulation of amiodarone. The pulmonary vascularization was increased, and there was small bilateral pleural effusion. In the liver, low-density nodules were identified, but their assessment was limited due to lack of contrast media imaging. In addition, there were sparse nodular calcifications in the liver parenchyma, whose density was diffusely increased (suggestive of amiodarone deposition). Dilation of the inferior vena cava and of the hepatic veins, as well as ectasia of the gallbladder, was observed. No lymph node enlargement was identified. The heart was diffusely enlarged, with predominance of the left atrium.

Because the patient was emaciated, had moderate reduction of the LVEF and altered pulmonary imaging, the search for consumptive syndrome causes other than heart failure began. Sputum tests (three samples on different days) were negative for acid-fast bacillus. The bacteriologic examination of the sputum revealed the usual local flora: Gram-positive bacilli, Gram-positive cocci, Gram-negative bacilli, Gram-negative diplococci and Gram-negative coccobacilli.

The patient was submitted to biopsy of the pulmonary nodule (October 12, 2016), which revealed a granulomatous chronic inflammatory process with extensive area of necrosis. The search for fungi was negative and that for acid-fast bacillus was ongoing.

On the night of October 12, 2016, 12 hours after the biopsy, the patient woke up complaining of ill-defined malaise. Her initial examination revealed arterial blood pressure of 50/40 mmHg, O_2_ saturation of 99% with O_2_ catheter, tachycardia and tachypnea. The patient had a cardiac arrest with pulseless electrical activity. She was resuscitated but developed asystole and died.

## Clinical aspects

This 60-year-old patient had the following problems: dyspnea, dry cough, weight loss, slightly elevated body temperature, anemia, enlarged pulmonary hila, hilar calcifications (lymph nodes) and pulmonary nodule (granuloma with necrosis). In addition, she had the following antecedents: Chagas disease (not confirmed, because no serology for Chagas disease was performed), systemic arterial hypertension, heart failure with reduced ejection fraction, and previous akinetic areas, apical aneurysm and sustained ventricular tachycardia.

Of the granulomatous diseases, sarcoidosis has unspecific symptoms, such as fever, weight loss, nocturnal sweating and fatigue. Other symptoms depend on the organs or parts of the body affected, such as the lungs (dry cough, dyspnea, chest pain), eyes (eye pain, blurred vision), skin, musculoskeletal system (joint pain, myalgias) and lymph nodes (swelling).^1^ Although heart involvement is diagnosed in 5% to 10% of the cases, on postmortem examination, it can range from 10% to 76%, causing bundle-branch block, repolarization disorders, arrhythmias and cardiomyopathy.^2,3^ Isolated cardiac sarcoidosis can occur in up to 25% of the cases; thus, absence of extracardiac sarcoidosis does not exclude heart involvement.^4,5^

The most common clinical characteristic of cardiac sarcoidosis is biventricular heart failure, with or without evidence of noncardiac involvement. In addition, mitral regurgitation can be severe and caused by the involvement of the papillary muscle, which could explain this patient's mitral regurgitation.^6^ Ventricular arrhythmias (sustained or non-sustained ventricular tachycardia and premature ventricular beats) are the second most common clinical presentation of cardiac sarcoidosis, occurring in approximately 30% of the cases.^7^

The echocardiographic findings in patients with cardiac sarcoidosis vary and can include focal areas of edema, resulting in wall thickening and hypertrophic cardiomyopathy mimicking (for example, asymmetric septal hypertrophy), or, in more advanced patterns of involvement, focal areas of akinesia, dyskinesia or aneurysm.^8^

Although cardiac sarcoidosis has been described as a restrictive cardiomyopathy, its most common phenotype is dilated cardiomyopathy, with occasional aneurysm formation.^6^

Because the above described disease has many findings in common with those of our patient, our clinical hypothesis is systemic sarcoidosis with cardiac and pulmonary involvement. However, infectious causes for the respiratory impairment cannot be ruled out, the most common and prevalent in Brazil being tuberculosis, which also forms granulomas.

It is worth noting, however, that sarcoid granulomas are usually "non-caseating", that is, have no necrosis. Thus, the lymph node biopsy of our patient does not support that diagnosis. (Gustavo Alonso Arduine, MD)

**Diagnostic hypothesis:** syndromic: heart failure with reduced ejection fraction; etiological: systemic sarcoidosis (cardiac and pulmonary). (Gustavo Alonso Arduine, MD)

**Final finding:** Mixed septic and cardiogenic shock (Gustavo Alonso Arduine, MD)

## Postmortem examination

The postmortem examination revealed an emaciated female patient, with a cutaneous sign of thoracic needle puncture on the right side. Chest opening evidenced blood clots on the parietal pleura and blood collection in the right pleural cavity (total of 1400 mL).

**Figure 8:**
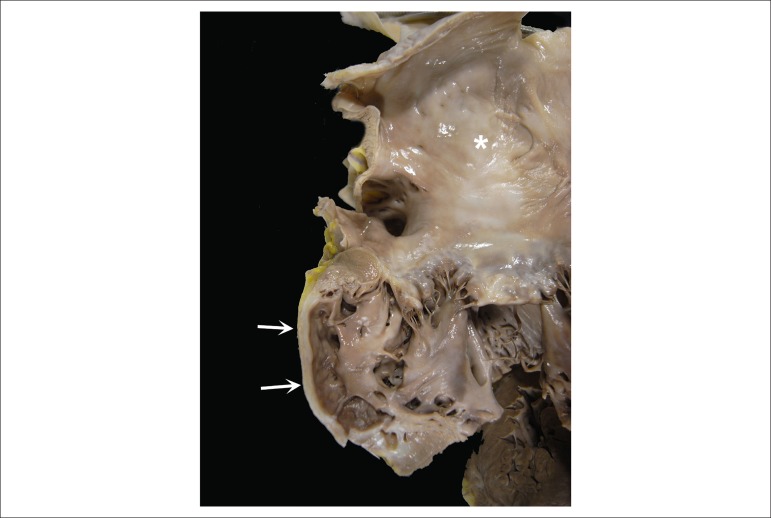
Gross examination: aneurysmal thinning of the left ventricular lateral wall, with fibrous replacement of the myocardium (arrows). The left atrium (asterisk) is extremely dilated.

**Figure 9:**
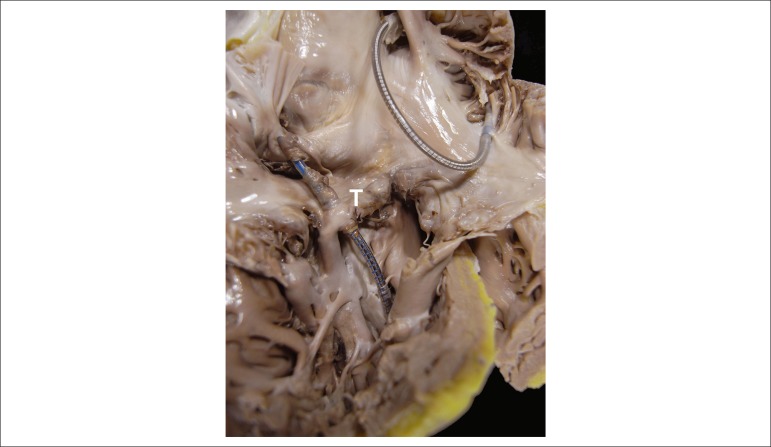
Right chambers showing two endocardial pacemaker metallic leads, one attached to the atrium and the other to the ventricular apex. T- Tricuspid valve.

The heart was moderately enlarged (450 g), with mild biventricular dilation and an aneurysm formation with thinning of the left lateral myocardial wall (approximately 4x3 cm) (Figure 8). That area evidenced fibrous replacement of the myocardium. The mitral valve showed signs of regurgitation secondary to annulus dilation. In the right chambers, the pacemaker metallic leads could be seen, one attached to the atrium and the other to the trabecular portion of the ventricle (Figure 9). On its way through the tricuspid valve, the lead was adhered and covered by a whitish sheath. No cavitary thrombus was seen.

**Figure 10:**
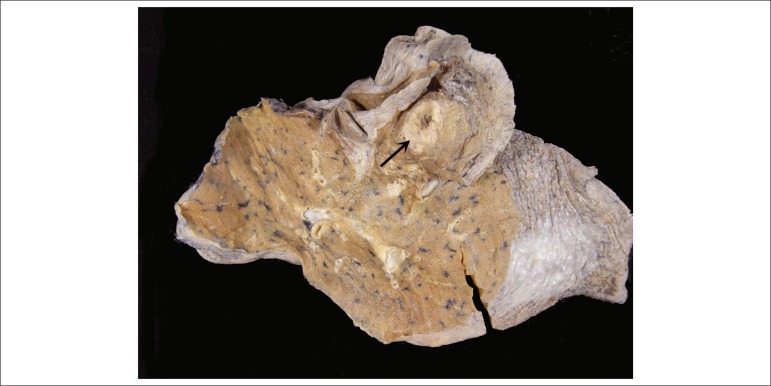
Cut surface of the lung showing an ill-defined nodule with necrotic center (arrow).

The examination of the lungs evidenced an ill-defined brownish nodule, with necrotic center, in the right middle lobe, measuring 2.5 cm in its long axis (Figure 10).

**Figure 11:**
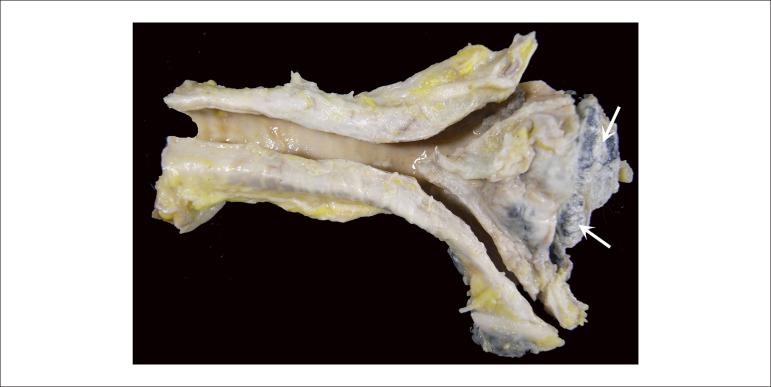
Gross examination of the longitudinally opened trachea on its posterior face. In the subcarinal region, confluent lymph nodes with whitish nodular areas are seen (arrows).

The hilar and subcarinal lymph nodes were enlarged, confluent, and had extensive nodular, whitish areas (Figure 11).

The liver weighed 2223 g and had a finely granular surface.

The microscopic study of the myocardium showed hypertrophied cardiomyocytes, varied focal fibrosis, and focal and mild inflammatory infiltrate.

**Figure 12:**
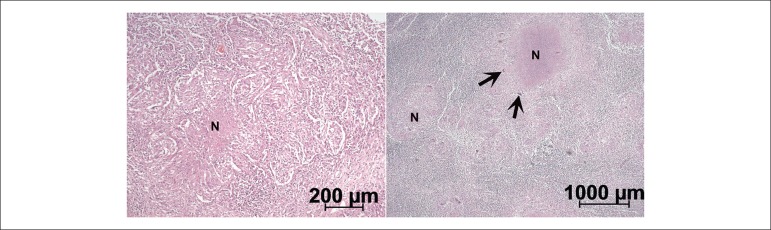
Microscopic examination: granulomatous chronic inflammation of the lung (left panel) and lymph node (right panel). Note the numerous giant cells (arrows) and foci of caseating necrosis (N). Hematoxylin-eosin, 10X and 5X.

The microscopic study of the lungs and lymph nodes showed extensive chronic granulomatous inflammation with caseating necrosis, including in the nodular area of the pulmonary parenchyma (Figure 12). The search for acid-fast bacilli was positive, with a small number of bacilli in the caseating lesions (not shown).

**Figure 13:**
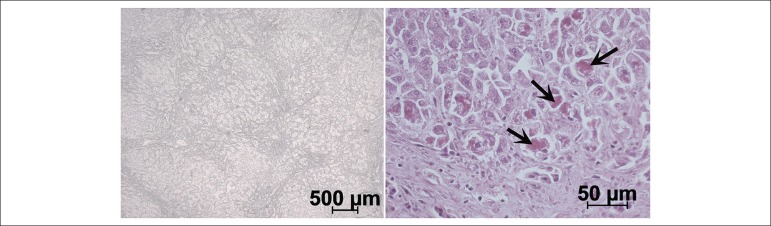
Photomicrograph of the liver. Left panel: nodular transformation of the parenchyma, reticulin stain, 5X. Right panel: hepatocytes with multiple eosinophilic bodies (Mallory bodies - arrows), hematoxylin-eosin, 40X.

The microscopic study of the liver evidenced diffuse nodular transformation, expansion of the portal spaces and diffusely damaged hepatocytes, characterized by the presence of multiple eosinophilic inclusions in their cytoplasm (Mallory bodies) (Figure 13). (Vera Demarchi Aiello, Prof., M.D.)

**Anatomopathological diagnoses:**
- Chronic heart disease, probably of Chagasic etiology, with an aneurysm of the lateral wall;
- Productive-caseating tuberculosis in the lungs and mediastinal lymph nodes;
- Chronic liver disease progressing to cirrhosis, with characteristics of cellular damage secondary to the chronic use of amiodarone;
- Hemothorax. (Vera Demarchi Aiello, Prof., M.D.)

## Comments

The patient had chronic heart disease with arrhythmia, having received specific treatment with pacemaker implantation and prescription of antiarrhythmic drugs. Her clinical condition worsened due to the development of productive-caseating tuberculosis in the lungs and mediastinal lymph nodes, which motivated the final diagnostic investigation. The clinical hypothesis of sarcoidosis was ruled out by the finding of the infectious agent (acid-fast bacilli) in the granulomatous lesions.

Despite the lack of diffuse myocarditis on the microscopic study, Chagasic heart disease is the most probable diagnosis, considering the gross morphological aspect of the heart, with fibrous replacement of the left ventricular lateral/inferior wall and the presence of diffuse interstitial fibrosis, although such findings are not characteristic.

The microscopic findings in the liver parenchyma point to a type of cell damage related to drug toxicity. Because of the report of this patient having received amiodarone during her disease, we concluded that her liver damage is related to that drug. That type of lesion is characterized by nodular transformation (cirrhosis) and hepatitis with several Mallory bodies. In the past, such bodies were known to be associated with alcoholic liver disease. However, several studies have shown the pathogenic role of amiodarone and its metabolites on the development of liver disease.^9,10^ Those substances accumulate in the hepatocytes, Kupffer cells and ductal cells, resulting in inhibition of the removal of lysosomal lipids. Hepatotoxicity occurs in 1% to 3% of the patients treated with amiodarone and seems to be dose-dependent (cumulative). However, the prevalence of pulmonary toxicity is higher, estimated at 5% to 7%. We believe that the liver damage contributed to the changes in coagulation that culminated in hemothorax.

There was no time to initiate the specific treatment against tuberculosis, which might have had a positive impact on this patient's outcome. (Vera Demarchi Aiello, Prof., M.D.)
